# Pterostilbeno Pós Infarto Agudo do Miocárdio: Efeito no Coração e Pulmão

**DOI:** 10.36660/abc.20211017

**Published:** 2022-02-14

**Authors:** Bruna Paola Murino Rafacho

**Affiliations:** 1 Universidade Federal de Mato Grosso do Sul Faculdade de Ciências Farmacêuticas, Alimentos e Nutrição Campo Grande MS Brasil Universidade Federal de Mato Grosso do Sul – Faculdade de Ciências Farmacêuticas, Alimentos e Nutrição,Campo Grande, MS – Brasil

**Keywords:** Pterostilbeno, Infarto Agudo do miocárdio, Estresse Oxidativo

As doenças cardiovasculares são a principal causa de morte no Brasil e no mundo.^1 , 2^ No Brasil, as doenças isquêmicas do coração ocupam a primeira causa de morte cardiovascular,^3^ tendo o infarto agudo do miocárdio taxa de mortalidade de 9,06% até outubro de 2021.^4^

A isquemia do miocárdio gera a liberação de espécies reativas de oxigênio (EROs) e de nitrogênio (ERNs) que, por sua vez, estimulam o aumento de mediadores pró-inflamatórios que ativam diferentes vias para promover a reparação da região infartada, processo conhecido como remodelação cardíaca.^5 - 7^ Entretanto, quando há produção prolongada de espécies reativas, incapacidade do sistema antioxidante e amplificação das alterações inflamatórias e metabólicas do coração, há comprometimento da região não infartada, levando a alterações progressivas na geometria ventricular.^8^ Progressivamente, ocorrem alterações nos ventrículos esquerdo e direito associadas a modificações hemodinâmicas nos vasos pulmonares e consequente hipertensão pulmonar.^5 , 9^ Neste contexto, a busca por tratamentos que atenuem as alterações cardíacas e pulmonares com foco no equilíbrio redox se destacam.

Nos cardiomiócitos, as espécies reativas podem ter origem nas enzimas nicotinamida adenina dinucleotíde (NADPH) oxidases, xantina oxidase e óxido nítrico sintase (NOS) desacoplada.^10^ A produção das especies reativas podem ser bloqueadas nos tecidos por meio do sistema antioxidante enzimático, que incluem as enzimas glutationa peroxidase (GPx), superóxido dismutase (SOD) e catalase (CAT), ou por sistemas não enzimáticos, que incluem glutationa, vitamina C e E.^6^ Recentemente, o uso de componentes antioxidantes derivados de alimentos tem se destacado em diferentes modelos. Os polifénois são um grupo de moléculas encontradas em alimentos vegetais e tem sido associados a efeitos benéficos no tratamento de várias condições clínicas.^7^ O pterostilbeno (PS) é um derivado do resveratrol encontrado em frutas vermelhas e uvas e com efeito antioxidante em diferentes modelos.^5 , 11 , 12^

Nesta edição dos Arquivos Brasileiros de Cardiologia, Tasca et al. mostram o efeito protetor deste composto nos tecidos cardíacos e pulmonares em modelo experimental de infarto agudo do miocárdio (IAM) em ratos Wistar. O IAM promoveu alterações funcionais cardíacas, acompanhadas de aumento das enzimas oxidantes (maior expressão de xantina oxidase, maior atividade da NADPH oxidase no ventrículo direito - VD) e menor concentração de antioxidantes (sulfidrilas e NOS). Nos pulmões, houve redução da concentração de SOD 14 dias após o evento isquêmico nos animais que não receberam o composto antioxidante. Em relação aos efeitos do PS, este reverteu as alterações encontradas nas enzimas antioxidantes do VD pós-infarto e aumentou a concentração de glutationa, SOD e CAT no tecido pulmonar, confirmando o efeito antioxidante. É interessante observar ainda que houve aumento da expressão do fator nuclear 2 relacionado ao eritroide 2 (Nrf2), um regulador chave da resposta antioxidante, indicando um potencial mecanismo de ação do PS.^13^

Desta forma, nota-se que a avaliação do tecido pulmonar e do VD após o IAM é um diferencial do trabalho, bem como a caracterização do efeito antioxidante do PS nesses tecidos, abrindo caminhos para melhor entendimento das ações de compostos antioxidantes nas alterações pós infarto e o uso do PS em diferentes tempos de intervenção e em outros modelos.
