# Supplementary material for: Pterostilbeno Pós Infarto Agudo do Miocárdio: Efeito no Coração e Pulmão
Source: Arq Bras Cardiol. 2022 Feb 14;118(2):446–7. [Article in Portuguese] doi: 10.36660/abc.20211017 (PMC8856697; doi:10.36660/abc.20211017)
Supplement: Translation

# Translation: Pterostilbene after Acute Myocardial Infarction: Effect on Heart and Lung Tissues

**Authors:** Bruna Paola Murino Rafacho

**Affiliations:** 1 Brazil Universidade Federal de Mato Grosso do Sul – Faculdade de Ciências Farmacêuticas, Alimentos e Nutrição, Campo Grande, MS – Brazil

**Keywords:** Pterostilbene, Acute Myocardial Infarction, Oxidative Stress

Cardiovascular diseases are the main cause of death worldwide.^1 , 2^ In Brazil, ischemic heart diseases are the leading cause of cardiovascular death,^3^ and acute myocardial infarction presented a mortality rate of 9.06% until October 2021.^4^

Myocardial ischemia generates reactive oxygen species (ROS) and reactive nitrogen species (RNS), which, in turn, increases pro-inflammatory mediators that might activate different pathways to promote repairing of the infarcted region, a process known as cardiac remodeling.^5 - 7^ However, when there is prolonged production of reactive species, incapacity of the antioxidant system and amplification of inflammatory and metabolic changes in the heart, non-infarcted region is impaired, leading to progressive changes in ventricular geometry.^8^ Progressively, changes can occur in the left and right ventricles associated with hemodynamic changes in the pulmonary vessels and consequent pulmonary hypertension.^5 , 9^ In this context, the search for treatments that might attenuate cardiac and pulmonary changes focusing on redox balance stand out.

Regarding cardiomyocytes, reactive species can be originated from nicotinamide adenine dinucleotide (NADPH) oxidases, xanthine oxidase and uncoupled nitric oxide synthase (NOS) enzymes.^10^ Reactive species production generated in tissues can be blocked through enzymatic antioxidant system, including glutathione peroxidase (GPx), superoxide dismutase (SOD) and catalase (CAT), or through non-enzymatic systems, which include glutathione, vitamin C and E.^6^ Recently, the use of antioxidant components derived from foods has been highlighted in different models. Polyphenols are a group of molecules found in plant foods and have been associated with beneficial effects in prevention and treatment of various clinical conditions.^7^ Pterostilbene (PS) is a resveratrol derivative found in red fruits and grapes and it has been exhibited antioxidant effect in different disease models.^5 , 11 , 12^

In the present issue of Arquivos Brasileiros de Cardiologia, Tasca et al. show the protective effect of this compound on cardiac and pulmonary tissues in an experimental model of acute myocardial infarction (AMI) in Wistar rats. AMI promoted cardiac functional alterations, accompanied by an increase in oxidizing enzymes (greater expression of xanthine oxidase, greater NADPH oxidase activity) and lower concentration of antioxidants (sulfhydryls and NOS) in the right ventricle (RV). In lung tissue, reduction in the concentration of SOD 14 days after the ischemic event in animals that did not receive the antioxidant compound was observed. Regarding PS effects, it reversed the changes found in antioxidant enzymes of the post-infarction RV and increased the concentration of glutathione, SOD and CAT in the lung tissue, confirming the antioxidant effect. Interestingly, there was an increase in the expression of nuclear factor 2 related to erythroid 2 (Nrf2), a key regulator of the antioxidant response, indicating a potential mechanism of action for PS.^13^

Thus, it is noted that the assessment of lung tissue and the RV after AMI is a differential of the present research, as well as the characterization of the antioxidant effect of PS in these tissues, opening paths for a better understanding of antioxidant compounds action in post-infarction changes and the use of PS in different times of intervention and other models.
